# Sex-Related Differences in the Trade-Off between Foraging and Vigilance in a Granivorous Forager

**DOI:** 10.1371/journal.pone.0101598

**Published:** 2014-07-01

**Authors:** Thibaut Powolny, Vincent Bretagnolle, Astrid Aguilar, Cyril Eraud

**Affiliations:** 1 Office National de la Chasse et de la Faune Sauvage, Villiers en bois, France; 2 Centre d'Etudes Biologiques de Chizé, Centre National de la Recherche Scientifique, Villiers en bois, France; University of Debrecen, Hungary

## Abstract

The relationship between intake rate and food density can provide the foundation for models that predict the spatiotemporal distribution of organisms across a range of resource densities. The functional response, describing the relationship between resource density and intake rate is often interpreted mechanistically as the relationships between times spend searching and handling. While several functional response models incorporate anti-predator vigilance (defined here as an interruption of feeding or some other activity to visually scan the environment, directed mainly towards detecting potential predators), the impacts of environmental factors influencing directly anti-predator vigilance remains unclear. We examined the combined effects of different scenarios of predation risk and food density on time allocation between foraging and anti-predator vigilance in a granivorous species. We experimentally exposed Skylarks to various cover heights and seed densities, and measured individual time budget and pecking and intake rates. Our results indicated that time devoted to different activities varied as a function of both seed density and cover height. Foraging time increased with seed density for all cover heights. Conversely, an increased cover height resulted in a decreased foraging time. Contrary to males, the decreased proportion of time spent foraging did not translate into a foraging disadvantage for females. When vegetation height was higher, females maintained similar pecking and intake rates compared to intermediate levels, while males consistently decreased their energy gain. This difference in anti-predator responses suggests a sexually mediated strategy in the food-safety trade-off: when resource density is high a females would adopt a camouflage strategy while an escape strategy would be adopted by males. In other words, males would leave risky-areas, whereas females would stay when resource density is high. Our results suggest that increased predation risk might generate sexually mediated behavioural responses that functional response models should perhaps better consider in the future.

## Introduction

The relationship between intake rate and food density can provide the foundation for models that predict the spatiotemporal distribution of organisms across a range of resource densities [1]. It has been previously demonstrated that the relationship between food density and feeding rate (*i.e*. functional response) results from differential time allocation between competing activities, such as searching, handling, and anti-predator vigilance [2]. Because anti-predator behaviour may monopolize a large amount of the foraging time-budget [2], [3], [4], anti-predator vigilance is often regarded as a major factor limiting feeding rates, and ultimately affecting the functional response. Paradoxically, though much work has been devoted to investigating individuals' responses to varying resource densities and predation risk independently [5], [6], only a few studies have experimentally considered the combined effects of these factors on foraging time-budget and energy gain [7], [8]. This could be important because food availability should impact patch selection by influencing intake rate, predator detection, and avoidance [9].

Changes in vigilant behaviour have been attributed to variation in predation risk, which is itself dependent on environmental factors including group-size for social or aggregative species [10], [11], [12], [13], predator type and/or abundance [14], distance to safe refuges [15], or predator detectability [16], [17]. Concerning this latter factor, many prey organisms can assess predation risk through cues signalling the presence of predators, either being auditory [18], chemosensory [19] or visual [20]. For prey species that rely on sight for detecting predators, vegetation structure may influence the perceived predation risk [11], and is therefore likely to affect foraging habitat selection [21] as well as anti-predator strategies. However, the effect of vegetation structure on vigilance is mixed, since reduced visibility caused by vegetation may increase vigilance [14], [16], [17], [22], or increase camouflage and thus reduce vigilance [23]. Similarly, depending on species and their anti-predator strategies, patches with complex vegetation structure may be assimilated as dangerous by increase the perceived risk of predation [6], [16], [24] or protective by limiting the prey detectability by predators [25], [26], [27].

Besides environmental factors, individual phenotypes may also modulate the foraging time-budget [10]. For instance, individuals in poor body condition were shown to have lower anti-predator vigilance and contrastingly, prioritize energy gain [28]. Sex is also expected as one crucial source of inter-individual variation in the optimization of foraging time-budget under varying food resource and predation risk. Indeed, predator's preferences and foraging tactics [29], [30] may result in sex-selective predation [31] with profound consequences on sex-specific anti-predator strategies. Several studies have found sex differences in the time allocated to anti-predator vigilance [32], [33]. However, the influence of sex on individual response to varying food resources and predation risk has been poorly studied, especially in avian models. Among birds, ground seed-eating species (such as many farmland birds, at least in winter) appear to be particularly exposed to predation, since they live in open habitats that increase the distance to safe refuges. In addition, although habitat use and diet in the breeding season have received considerable attention [34], [35], habitat use and foraging behaviours during winter were less investigated [21], [36], [37], [38].

In this study we use an experimental approach and examine foraging behaviour of a ground seed eating farmland bird, the Eurasian Skylark *Alauda arvensis*, on artificial stubble substrates. Stubble fields are important foraging habitats for many farmland and granivorous bird species wintering in Europe [36], [39], but the effects of habitat structure on foraging and anti-predator strategies of these species remain unclear [6], [7], [37]. Although vigilance time is strongly influenced by predation risk [39], only few studies have directly investigated the combined effects of resource density and the perceived predation risk on functional response [7]. In order to investigate how the trade-off between foraging and vigilance is altered, we experimentally increased the visual obstruction from predators by exposing birds to three different vegetation heights. In addition to the predation risk, we manipulated resource availability on experimental patches. Theory predicts a differential response in terms of vigilance under contrasting resource availability, but several additional confounding factors may further balance this relationship such as competition or scrounging [40]. In some bird species, annual survival rates were found to be higher in males than in females, probably due to differential breeding costs [41] or sex-differences in risk-taking behaviour under foraging circumstances [42]. Hence males and females could behave differently with regard to their degree of exposure to predation and their differential survival prospects. If so, we predict that birds might differentially adapt their vigilance in response to the perceived predation risk according to resource density, but also their sex.

## Methods

### Ethics Statement

This work was performed with governmental authorizations from the Préfecture des Deux-Sèvres (Niort, France, no. 10.79-219). All experiments were carried out in compliance with French legal requirements. This study was carried out in strict accordance with the recommendations in the Guide for the Care and Use of Laboratory Animals of the National Institutes of Research. The protocol was approved by the Committee on the Ethics of Animal Experiments of the National Conservation Authority (permit no. 79349). Among all catching birds, animals showing any sign of sickness were removed before entering the experiment (*N* = 3). Bird captures were performed under permit from the National Hunting and Wildlife Agency to TP (no. 2009-014). All experiments were conform to the guidelines for the treatments and use of animals in behavioural research and teaching as published by the Association for the Study of Animal Behaviour (ASAB 2012).

### Study species

The skylark is a common species of open farmland habitats throughout Europe [43] that suffers a sustained and often severe decline in relation to changes in agricultural practices since the last few decades [44]. Being strongly territorial and feeding on insects in spring and summer, the species shows a marked gregarious behaviour on migratory stop-over and wintering quarters where birds live in groups of various sizes. Outside the breeding season, the species mainly feeds on seeds gleaned on the ground and vegetative parts of plants. The species shows a slight sexual dimorphism, with males being on average larger than females [43].

### Bird capture and maintenance

We caught skylarks along the Atlantic flyway during the post-nuptial migration, between October and November 2010. Upon capture, the birds were colour ringed, weighed (precision: ±0.1 g; males: 41±0.2 g; females: 31.7±0.22 g) and their tarsus and maximum wing chords were measured. Blood samples were taken, and their sex was obtained from molecular analysis (see [45] for details). Thereafter, birds were randomly assigned to groups of 10 to 12 individuals and acclimatized for two months in 4*3*2 m (L*W*H) outdoor aviaries at the Centre d'Etudes Biologiques de Chizé (CEBC-CNRS, Western France). Birds were fed *ad libitum* with a commercial seed mixture, grit, green material (i.e. oilseed rape) and tap water. Food was dispensed on a 2 m∧2 synthetic green turf (height: 1 cm; density: 12 blades/cm∧2) to accustom individuals to the experimental set-up. To limit the time spent under captive conditions all birds were released into the wild in the middle of March, during the pre-nuptial migration.

### General experimental procedures

All experiments were conducted from the 7^th^ February to 4^th^ March 2011. The night before each trial, all focal birds were weighed and deprived from food (between 12 and 16 hours) until the next morning. Trials were only performed from 8:00 to 12:00 am in order to avoid too long fasting periods. We did not conduct experiments on rainy or excessively windy days, as these extreme weather conditions would likely affect visibility and foraging behaviour. All trials were carried out in a separate outdoor aviary, showing identical proportions and layout to the housing aviaries.

We experimentally exposed birds to contrasting seed densities (3 levels) and vegetation heights (3 levels). Each bird was exposed to all of the 9 treatment combinations in a random order during the experiment. The experimental arena consisted of a 50×50×40 cm (H) wire cage (mesh size: 1×1 cm) enabling visual contact amongst birds. The cage was placed over an artificial green turf substrate surrounded by stubble. To mimic natural stubble habitat, we attached cereal straw to a brown polystyrene board (total width around the cage: 34 cm). The straw was arranged in rows 12 cm apart at a density of 110 straws/m, which was equivalent to the density measured in natural stubble fields around our laboratory (*N* = 12; mean ± SE; 12.4±2.21 cm; 110±7 straws/m). To investigate the influence of the visual obstruction on foraging activities, we used three different stubble heights: 0 cm (no stubble), 15 cm (comparable to the height measured in 20 randomly selected patches in 5 stubble fields (mean ± SE = 15.2±1.87 cm; range 9.8–17.3) and finally 35 cm in height. To investigate the influence of seed density on foraging activities, we used millet seeds (*Panicum miliaceum*) owing to their homogeneity in both colour (white) and size (mass = 0.007±0.0003 g (mean ± SD); sample size: 300 seeds). Birds were exposed to three different seeds densities (i.e. 100, 400 and 1600 seeds.m^−2^, counted by hand) randomly scattered on the artificial green turf substrate. All these densities fell within the range of seed densities recorded in arable fields [46], [47].

Among the birds captured during the fall migration, 10 females and 10 males were randomly selected and tested for all treatment combinations (i.e. 3 vegetation heights ×3 seed densities) with no replicate. During winter, skylarks adopt particular aggregative strategies and restrict foraging behaviour in the absence of conspecifics [48]. Hence, one non-focal bird (different from the 20 tested birds) was randomly assigned to each trial, and individually kept in identical wire mesh cages at a distance of 1 m from the focal individual. To avoid synchrony in behaviour [49], non-focal birds were not provided with food during tests. Group composition and consequently group sex-ratio, including the focal individual and the con-specific (non-focal bird) thus varied randomly from test to test to avoid systematic association between partner birds and experimental treatments or conditions. There were between six and eight trials per day, but neither the focal nor the non-focal birds experienced more than one trial in any one day. Ten minutes before each trial, the birds were placed in their respective cages so that they became accustomed to the experimental set-up. Each trial lasted five minutes, starting when the first peck was recorded. Focal birds were video-recorded using a camcorder set 1 m above the ground and about 1 m from the focal bird.

### Data collection and analysis

The behaviour of focal birds was analysed from videotapes using an event-recording program (EthoLog 2.2 [50]). All videos (*n* = 180) were analysed at 1X reading speed after testing on a subset of trials that this speed provided correlated results to those obtained by analyzing the videos frame by frame. (Foraging time; Pearson correlation coefficient: *r* = 0.781; *p*<0.01, *n* = 90).

Both the pecking rate (expressed as the number of pecks during the 5 minutes trial) and the time devoted to foraging and vigilance (in seconds) were separately quantified. Using video recording, the posture of birds was analysed for each trial. Following Whittingham & Markland [51] and Powolny et al. [48], a bird was considered to be vigilant when its head was above the horizontal line made by its body, and not orientated towards the ground. Conversely, a bird was considered as foraging when its head was below the horizontal line and when it actively scanned the ground or pecked. In addition to these criteria, we used the position of the bill (oriented towards the ground or staying horizontally or towards the sky) to discriminate behaviours implied in foraging and vigilant activities. The handling time - i.e. the time needed to consume one food item - and vigilance could not be separated given that skylarks do not handle millet seeds [48]. However, although head down posture is associated with foraging behaviour, this position still may allow vigilance [52]. Thus, we added an opaque band of 5 cm high around the bottom of the cage leading to no possibility of looking out the cage when the bird's posture was considered as foraging. So, given that vigilance and foraging represented the only two postures recorded in trials and because they were two mutually exclusive activities in our case study, only the time spent foraging was considered in analyses. Additionally, we quantified the time spent moving for a density of 100 seeds.m^−2^ and 400 seeds.m^−2^, which correspond to the seed density commonly measured on stubble fields [47], and 1600 seeds.m^−2^ representing our non limiting density. We paid particular attention to displacement under vigilant posture. Vigilant skylarks were considered as moving when they scanned their environment while walking with their head up.

The use of green turf allowed us to collect all remaining seeds. At the end of each trial, seeds remaining on the green turf were counted by hand by a single person without information about trials condition (sex, seed density or vegetation height). The corresponding values were used to calculate food intake rate (expressed as the number of seeds consumed during the 5 minutes of trial).

We used General Linear Mixed Models (GLMMs) to investigate the effects of seeds density, vegetation height and sex of the focal bird on time spent foraging, pecking and intake rates and time spent moving. Because focal individuals were tested for all treatment combinations, models were fitted with bird identity as a random factor and nested within sex. Analyses were separately performed for each dependent variable and followed a backward selection procedure starting from a full model that included all factors, their two-way interactions and a set of continuous covariates designed to control for the conditions in which the tests were conducted. These covariates were (Table 1): date, minimum nightly temperature preceding each trial, body mass and fasting duration (corresponding to the time since the sunrise). In addition, the effect of the sex of the conspecific (i.e. the non-focal bird) was also considered both as a main effect and in interaction with the sex of the focal bird. Starting by continuous covariates aiming to control for trial conditions, non-significant terms at *P*≤0.05 were sequentially removed to achieve a minimal adequate model [53]. Wherever an interaction term was left in a model, the two corresponding factors were also included as main effects. Post-hoc comparisons were performed using Tukey's HSD tests. Both foraging and moving times were log-transformed while both intake and pecking rates were square-root transformed to ensure normality and homoscedastcity assumptions.

**Table 1:** Explanatory and response variables entered into models for the foraging time, intake and pecking rates studies.

Variable	Factor/covariate	Notes
**Date**	co-variate	julian date (1 for 1^st^ January)
**Minimum temperature**	co-variate	minimum temperature the night before being tested
**Body mass**	co-variate	body mass the night before being tested
**Focal bird sex**	factor	male or female
**Conspecific sex**	factor	male or female
**Food deprivation**	co-variate	in minutes
**Seed density**	factor	100; 400 or 1600 seeds/m^2^
**Cover height**	factor	0; 15 or 35 cm
**Foraging time**		focal bird searching for food (based on head position); in seconds
**Intake rate**		number of seeds consumed during test
**Pecking rate**		number of pecks during test

To test for habituation, GLMMs were performed on pecking and intake rates and foraging time using trial number as continuous covariate. Results did not show any evidence for habituation (pecking rate: *F* = 1.633; *df* = 1, 173; *p* = 0.204; intake rate: *F* = 0.048; *df* = 1, 173; *p* = 0.827; foraging time: *F* = 0.548; *df* = 1, 173; *p* = 0.862). All analyses were performed using SPSS 17.0 software. Means are expressed ± SE.

## Results

We found no evidence that the foraging behaviour of birds varied according to date or the fasting duration since these covariates were systematically removed during model selection (Table 2). Conversely, body mass significantly affected pecking, but not intake rates and time of foraging or movement. An effect of the ambient temperature experienced the night that preceded the trials suggested varying individuals' needs in relation to environmental conditions: the time spent in foraging, the number of pecks and ultimately intake rate, all increased as the minimum nightly temperature decreased (Table 2).

**Table 2:** Fixed effects explaining variation in time allocated to foraging, pecking and intake rates and the time spent in movement under vigilant posture.

Dependent variable	Fixed effect	Estimates	Df/DfDen	*F* values	*P*	*r^2^*
**Foraging time**	minimum temperature	−0.02	1, 150	5.71	0.018	0.538
	focal bird sex	*female*:0.06	1, 18	0.63	0.434	
	conspecific sex	*female*:−0.28	1, 152	4.93	0.028	
	seed density	*100*∶−0.14	2, 149	14.36	<0.0001	
		*400*∶−0.14				
	cover height	*0*∶0.3	2, 149	15.96	<0.0001	
		*15*∶0.21				
	focal bird sex x cover height		2, 149	3.39	0.036	
	focal bird sex x seed density		2, 149	3.54	0.031	
	focal bird sex x conspecific sex		1, 152	10.1	0.002	
**Pecking rate**	minimum temperature	−1.23	1, 151	8.83	0.003	0.591
	body mass	−1.78	1, 150	4.96	0.027	
	conspecific sex	female:0.44	1, 152	0.52	0.48	
	focal bird sex	*female*:−1.12	1, 18	11.39	0.001	
	seed density	*100*∶−1.79	2, 150	25.75	<0.0001	
		*400*∶−0.71				
	cover height	*0*∶1.55	2, 150	5.36	0.006	
		*15*∶1.51				
	focal bird sex x cover height		2, 150	6.97	0.002	
**Intake rate**	minimum temperature	−0.13	1, 151	25.65	<0.0001	0.726
	focal bird sex	*female*:1.54	1, 18	6.95	0.017	
	seed density	*100*∶−2.47	2, 153	130.22	<0.0001	
		*400*∶−1.18				
	cover height	*0*∶1.44	2, 153	20.48	<0.0001	
		*15*∶1.02				
	focal bird sex x cover height		2, 153	8.07	0.001	
**Time in movement**	focal bird sex	*female*:0.78	1, 18	13.45	0.004	0.618
	cover height	*0*∶1.01	2, 156	9.22	<0.0001	
		*15*∶0.56				
	focal bird sex x cover height		2, 156	7.08	0.001	

A final model was backward-selected using a linear mixed effect model. The variation observed in the df's comes probably from approximations that are made for the calculation of the random effect. In fact, we used 20 individuals, with 9 trials for each bird. This small difference is commonly observed when the number of individuals is not so high.

### Effects of seed density and cover height

**Figure 1:**
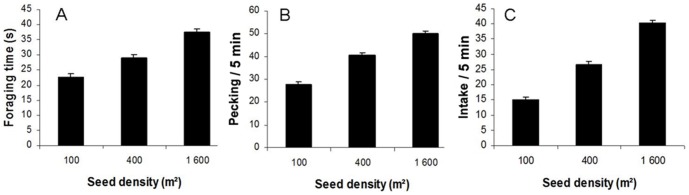
Effect of seed density on foraging time (A), pecking rate (B) and intake rate (C). Untransformed data are presented in the figure.

**Figure 2:**
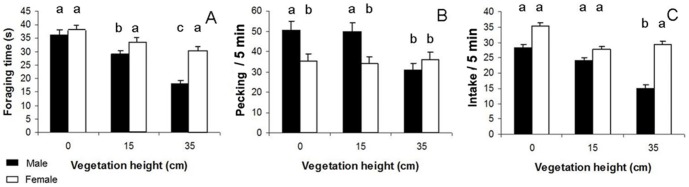
Cover height effect on foraging time (A), pecking rate (B) and intake rate (C). Black histograms represent males and white histograms represent females. Bar labelled with different letters are significantly different (p≤0.005).

All dependent variables describing foraging behaviour and performance enhanced with increasing seed density (Table 2). Individuals foraging in a patch with the lowest seed density treatment (i.e. 100 seeds.m^−2^) spent the shortest time foraging and had the lowest pecking and intake rates. Conversely, skylarks foraging in the richest patch (i.e. 1 600 seeds.m^−2^) spent the longest time foraging and had both the highest pecking and intake rates (Fig. 1). Intermediate values were recorded at medium initial seed density (i.e. 400 seeds.m^−2^). Skylarks foraging in a patch not surrounded by vegetation spent longer time foraging and had higher pecking and intake rates than those foraging in the highest cover height (i.e. 35 cm, Fig. 2). The interaction term between seed density and cover height was systematically removed during the selection process (Table 2) indicating that these two factors impacted foraging behaviour and performance in an additive way.

### Sex-related differences

**Figure 3:**
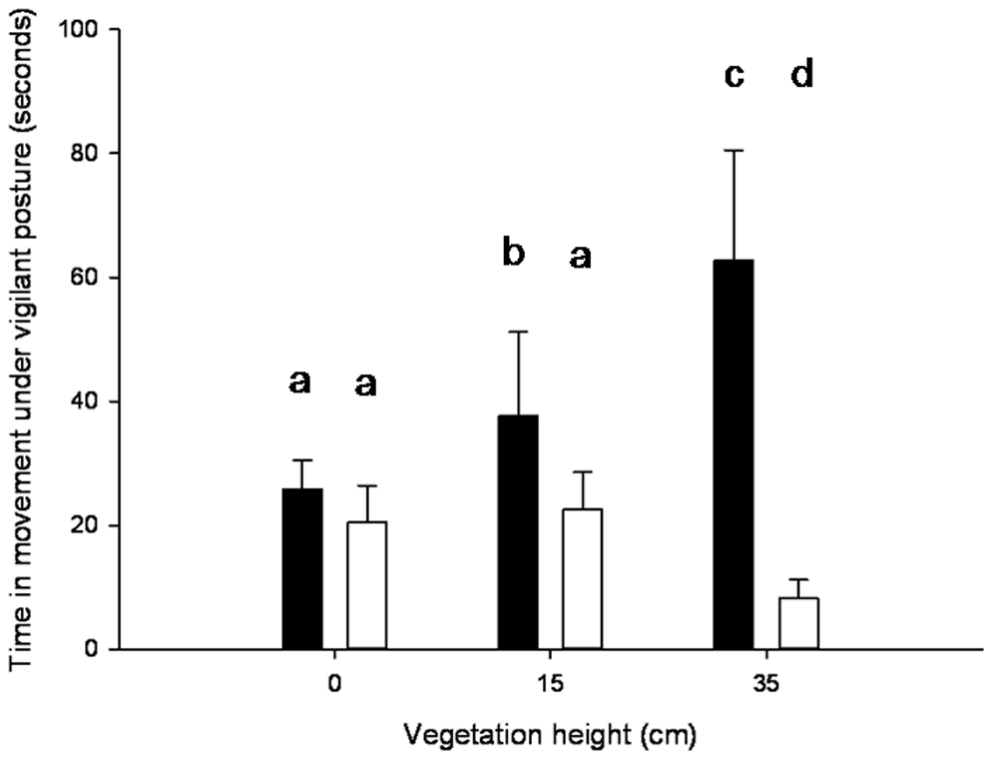
Average time (seconds) spent moving under vigilant posture. Black histograms represent males and white histograms represent females. Only results obtained for a seed density of 1600/m^2^ were representing. Vertical bars show standard errors. Bare labelled with different letters are significantly different (p≤0.005).

**Figure 4:**
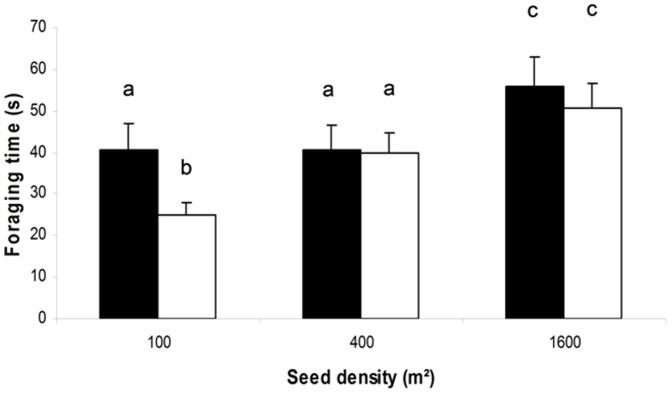
Effect of seed density on foraging time. Black histograms represent males and white histograms represent females. Vertical bars show standard errors. Bare labelled with different letters are significantly different (p≤0.005).

We found evidence that the effect of vegetation height on time spent foraging, pecking and intake rates differed significantly between sexes. More importantly, the interaction term between sex and cover height was retained in all final models (Table 2). The main component of this interaction was the greater sensitivity of males to the increase in vegetation height. With respect to the time budget, males reduced their time spent foraging at 35 cm in comparison to a cover height of 0 cm (post-hoc HSD Tukey: *p* = 0.001), conversely to females which allocated a similar amount of time at 35 cm and 15 cm (post-hoc HSD Tukey: *p* = 0.13; Fig. 2A) and between 0 and 35 cm (post-hoc HSD Tukey: *p* = 0.112; Fig. 2A). A similar pattern was observed for pecking rate: males reduced their pecking rate when the vegetation height dropped from 15 to 35 cm (post-hoc HSD Tukey: *p* = 0.03; Fig. 2B), whereas females preserved an almost identical pecking rate (post-hoc HSD Tukey: *p* = 0.970; Fig. 2B). Interestingly, male behaviour changed markedly when the vegetation height increased, increasing the time spent in movement (while adopting a vigilant posture). Contrary to males, females showed a decrease in time spent in movement (Fig. 3; Table 2). When seed density was highest (i.e. 1 600 seeds.m^−2^), males actually moved more than females at two of the three cover heights (0 cm: post-hoc HSD Tukey: *p* = 0.23; 15 cm: post-hoc HSD Tukey: *p* = 0.05; 35 cm: post-hoc HSD Tukey: *p* = 0.001; Fig. 3). As a consequence, only males' intake rate, but not females, was significantly reduced at 35 cm (post-hoc HSD Tukey: respectively: *p* = 0.003 and *p* = 0.271; Fig. 2C). While seed density and sex also interacted significantly in determining the allocation of time spent foraging (Table 2; Fig. 4), both sexes spent the shortest time foraging at low seed density and the longest time at the highest seed density (*LSMeans (±SE)*, females: 21.87±1.20 *vs*. 42.66±1.20; males: 23.99±1.20 *vs*. 33.11±1.20). While foraging time did not differ between sexes for the two highest seed densities (post-hoc HSD Tukey, 400 seed/m∧2: *p* = 0.87; 1600 seed/m∧2: *p* = 0.24) males spent more time under foraging posture at 100 seed/m∧2 than females (post-hoc HSD Tukey: *p* = 0.03; Fig. 4).

In addition, we found that the foraging behaviour of the focal birds depended on whether the conspecific non-focal bird was a male or a female (Table 2). For instance, pecking rate was significantly higher when conspecifics were males (*LSMeans (±SE)*, males: 46.37±4.69, females: 32.41±6.07). Even more surprisingly, there was a significant interaction between the sex of focal and non-focal birds on the time devoted to foraging (Table 2): males reduced their time spent foraging when conspecifics were females (*LSMeans (±SE)*, female non-focal bird: 19.05±1.23 *vs*. male non-focal bird: 37.15±1.17) while females were insensitive to the sex of conspecifics (*LSMeans (±SE)*, female non-focal bird: 33.88±1.23 *vs*. male non-focal bird: 30.2±1.17). Conversely, the sex of conspecifics had no significant effect on intake rate.

## Discussion

### Impacts of the vegetation height

In this study, we experimentally examined how resource availability and perceived predation risk affected time budget, pecking rate and ultimately seed intake in skylarks. As expected, our results indicated that time devoted to foraging varied both as a function of seed density and vegetation height. An increase in vegetation height consistently affected time-budget in skylark and resulted in a decrease of foraging time, probably through increasing vigilance. The reduced feeding time for the benefit of vigilance at the maximum vegetation height (i.e. 35 cm) indicated that visual obstruction would be perceived as a high predation risk in this species, especially in males, despite its obvious protective nature. Many studies have reported similar changes in time budget in obstructive habitat [10], [16], [17], [22], attesting that perceived predation risk was increased in our obstructive treatment. This result agrees with Verdolin [54], who suggested that habitat structure, particularly vegetation height, may significantly increase an animal's perceived predation risk, and strongly affects foraging behaviour. Natural vegetation and sometimes foraging substrate combine both protective and obstructive properties [26], [48], depending on the predator type and anti-predator strategies adopted by a species [26]. Increased vigilance under high cover height can also be explained by the additional utility of scanning when the view is obstructed. This anti-predator response can be related to the group-living strategy adopted by this species outside the breeding season. Skylarks are highly gregarious both during winter and migration, hence vigilance time in skylarks is unsurprisingly related to group-size [48]. It is worth noting that our current results are opposed to the predictions that could be made from the studies of Butler *et al*. [21] and Whittingham *et al*. [37]. In these two studies, authors have shown that the abundance of skylarks in winter was higher on control and untouched stubbles (12–17 cm high) than on experimentally left reduced stubbles (5–6 cm high). This treatment strongly impacted vegetation height (5–6 cm *vs*. 12–17 cm for topped and control plots, respectively) but had no effect on the soil seed density, leading to the conclusion that skylarks would associate higher predation risk with short stubbles [21], [37]. However, skylarks do not exclusively search for seeds on the soil surface but also peck at weed leaves and seed heads [43], which are likely to have been sharply reduced by topping. Hence, the hypothesis that topping had no effect on the potential energetic gain associated with short stubbles cannot be fully excluded as this treatment might have caused a reduction in food resources for the species.

### Impacts of the seed density

We found a positive relationship between foraging time and seed density independently of experimental level of visual obstruction, indicating that for each tested cover height, birds foraged more at high resource density. However, the benefits linked to the high seed density were balanced by the vegetation height, with a similar time spent foraging at a density of 100 seeds.m^−2^ without vegetation and at a density of 1600 seeds.m^−2^ with a vegetation height of 35 cm. This latter result is in accordance with Butler et al. [7] who investigated pecking rate in chaffinches, another seed eating bird. The relationship between food density and foraging rate showed that skylarks spent less time foraging at low resource densities, in agreement with previous studies on granivorous birds [55], [56]. However, the prediction that foraging should increase with food density does not appear to be consistent in all species. In his review regarding the effect of food density on vigilance and foraging in bird species, Beauchamp [40] reported a positive relationship between food density and vigilance time in 30% of cases, negative in 26% and an absence of relationship in 44% of cases. These contrasting results are probably due to several ecological factors modulating the effect of food density on vigilance such as spatial distribution of group, group size, handling posture or competition [40].

### Sex differences

In skylarks, male and female foraging time differed at a cover height of 35 cm (the more obstructive situations). While female skylarks maintained equal foraging time between the three cover heights, males exhibited a marked decrease in foraging time between the intermediate and the highest heights (15 and 35 cm, respectively. Several studies have shown that sex is an important determinant of individual foraging and vigilance levels [57], [58], reflecting differences in vulnerability to predation [59] and in the social factors that motivate scanning behaviour [60]. We observed intake differences between sexes in response to increased visual obstruction. In males, the decreased foraging time with vegetation height caused a reduction in the number of seeds consumed whereas females maintained their intake rate at the highest level of visual obstruction. In this later condition, our results also revealed an important contrast in the modality of vigilance adopted by each sex. While females tended to be immobile during vigilance sequences - suggesting a hiding or camouflage strategy-, males increased their time spent in movement – suggesting an escape strategy. Overall, these differences suggest a sex-related difference in the food-safety trade-off. Thus, males seem to prefer leave a risky-area, whereas females stay when resource density is high. As predation risk may vary among habitats, animals may not necessarily select habitats based solely on potential energetic gain. Consequently, individuals are likely to accept lower energetic benefits in order to forage in safer habitats [61]. Several field observational studies have also shown food-safety trade-offs, and differences between individual classes were observed [62]. For instance, Heithaus and Dill [63] suggested that juvenile male dolphins (*Tursiops aduncus*) are more willing to accept higher predation risk to achieve higher energy intake rates. A similar age class difference in risk-taking behaviour while foraging has been found in the redshank (*Tringa tetanus*) where juveniles attempt to maximize energy intake by foraging in high-risk areas [64]. In addition to age-classes, sex was also identified as a cause of behavioural differences in another bird species, the Western Sandpipers (*Calidris mauri*) where females were overrepresented in more dangerous habitats [65]. Our results suggest that female skylarks may also gain fitness benefits from risk-taking. By foraging in the energetically profitable habitats, females may increase their body-condition and their long-term survival while males tend to maximize escape and consequently immediate survival. Moreover, the energetic needs of sexes may be different. Females need more energy to be prepared for the egg laying hence they risk more to get the necessary energy than males do. To test for effective food-safety trade-off in skylarks, further experiments involving patch choices between males and females would be needed. As example, following the set-up proposed by Butler et al. [7] adapted to test males *versus* females skylarks, we may hypothesize that females should spent more time in a risky-environment than males if the energetic return can be important. One possible explanation of this pattern is that while we observed no difference in body condition between sexes, energetic needs to prepare the next pre-nuptial migration and breeding might differ between sexes.

In addition to resource availability and vegetation height, two other factors influenced foraging time allocation in skylarks. First, the minimum temperature during the night before experiments affected both the time spent under foraging activities and intake rate. Low temperature influences energy expenditure, through increased demand for thermoregulation, resulting in an increase in food intake or a higher proportion of time spent in foraging. This result is consistent with studies demonstrating the relationships between the maintenance of body mass and the increased food intake in response to thermal stress [66]. Second, the sex of the conspecific individual significantly affected focal bird foraging time and pecking rate. This result agrees with studies suggesting that vigilance time may be influenced by the sexual composition of the group for aggregative species [67]. Particularly, in our study, when conspecific were males, the pecking rate of focal bird increased but there was no effect on intake rate. This surprising result may highlight a decrease in foraging success (measured as intake rate/pecking rate) especially when focal and non-focal birds are males suggesting possible foraging competition by interference [55]. Another possible explanation for this intriguing result is the potential competition for mating, even if our experiment were done before the pre-nuptial migratory period.

In summary, our study showed that lower vegetation and high seed density enhanced foraging rate and food intake in our study model. It has been demonstrated [68] that perceived predation risk can alter habitat choice, which has been shown to influence survival and community structure [69]. Thus, habitat structure appears to be a major component of population dynamics, especially for community living in homogenous landscapes, such as farmland birds. Our results highlight that phenotypic traits such as sex, can generate variations in behavioural responses outside the breeding season, independently of body condition. A mechanistic understanding of such intraspecific variations in behaviour is critical to develop adequate functional models and may be relevant for management and conservation issue as well.

## References

[1] Sutherland WJ (1996) From individual behaviour to population ecology. Oxford University Press, Oxford.

[2] Baker DJ, Stillman RA, Smith BM, Bullock JM, Norris KJ (2010) Vigilance and the functional response of granivorous foragers. Funct Ecol 24: 1281–1290.

[3] Treves A (2000) Theory and method in studies of vigilance and aggregation. Anim Behav 60: 711–722. 11124869 10.1006/anbe.2000.1528

[4] Fernandez-Juricic E, Beauchamp G (2008) An experimental analysis of spatial position effects on foraging and vigilance in brown-headed cowbirds flock. Ethology 114: 105–114.

[5] Bednekoff PA, Lima SL (2005) Testing for peripheral vigilance: do birds value what they see when not overtly vigilant? Anim Behav 69: 1165–1171.

[6] Devereux CL, Whittingham MJ, Fernandez-Juricic E, Vickery JA, Krebs JR (2006) Predator detection and avoidance by starlings under differing scenarios of predation risk. Behav Ecol 17: 303–309.

[7] Butler SJ, Whittingham MJ, Quinn JL, Cresswell W (2005) Quantifying the interaction between food density and habitat structure in determining patch selection. Anim Behav 69: 337–343.

[8] Baker DJ, Stillman RA, Bullock JM (2009) The effect of habitat complexity on functional response of a seed-eating passerine. Ibis 151: 547–558.

[9] Cresswell W, Quinn JL, Whittingham MJ, Butler S (2003) Good foragers can also be a good detecting predators. Proc R Soc Lond B 270: 1069–1976. 10.1098/rspb.2003.2353 PMC1691342 12803897

[10] Elgar MA (1989) Predator vigilance and group size in mammals and birds: a critical review of the empirical evidence. Biol Review 64: 13–33. 10.1111/j.1469-185x.1989.tb00636.x 2655726

[11] Lima SL, Dill LM (1990) Behavioral decisions made under the risk of predation. A review and prospectus. Can J Zool 68: 619–640.

[12] Lima SL (1990) The influence of models on the interpretation of vigilance. In: Interpretation and Explanation in the study of Animal Behaviour: vol.2. Explanation, Evolution and Adaptation (Ed. By M.Bekoff and D. Jamieson), pp. 246–267. Boulder, Colorado: Westview Press.

[13] Krause J, Ruxton GD (2002) Living in groups. Oxford University Press, Oxford.

[14] Cresswell W (1994a) Flocking is an affective anti-predation strategy in redshank, Tringa tetanus. Anim Behav 47: 433–442.

[15] Frid A (1997) Vigilance by female Dall's sheep: interactions between predation risk factors. Anim Behav 53: 799–808.

[16] Metcalfe NB (1984) The effect of habitat on the vigilance of shorebirds: is visibility important? Anim Behav 32: 981–985.

[17] Arenz CL, Leger CW (1997a) Artificial visual obstruction, antipredator vigilance, and predator detection in the thirteen-lined ground squirrel (Spermophilus tridecemlineatus). Behaviour 134: 1101–1114.

[18] Berger J (1999) Anthropogenic extinction of top carnivores and interspecific animal behaviour: implications of the rapid decoupling of a web involving wolves, bears, moose and ravens. Proc R Soc Lond B 266: 2261–2267. 10.1098/rspb.1999.0917 PMC1690453 10629976

[19] Van Buskirk J, McCollum SA (1999) Plasticity and selection explain variation in tadpole phenotype between ponds with different predator composition. Oikos 85: 31–39.

[20] McGowan KJ, Woolfenden GE (1989) A sentinel sytem in the Florida Scrub Jay. Anim Behav 37: 1000–1006.

[21] Butler SJ, Bradbury RB, Whittingham MJ (2005) Stubble height affects the use of stubble fields by farmland birds. J App Ecol 42: 469–476.

[22] Arenz CL, Leger CW (1997b) The antipredator vigilance of adult and juvenile thirteen-lined ground squirrels (Sciuridae: Spermophilus tridecemlineatus): Visual obstruction and simulated hawk attacks. Ethology 103: 945–953.

[23] Lazarus, Symond M (1992) Contrasting effects of protective and obstructive cover on avian vigilance. Anim Behav 43: 519–521.

[24] Sharpe PB, van Horne B (1998) Influence of habitat on behaviour of Townsend's ground squirrels (Spermophilus townsendii). J Mammal 79: 906–918.

[25] Ekman J (1987) Exposure and time use in willow tit flocks: the cost of subordination. Anim Behav 35: 445–452.

[26] Lima SL (1987) Vigilance while feeding and its relation to the risk of predation. J Theor Biol 124: 303–316.

[27] Griesser M, Nystrand M (2009) Vigilance and predation of forest-living bird species depend on large-scale habitat structure. Behav Ecol 20: 709–715.

[28] Arenz CL, Leger CW (2000) Antipredator vigilance of juvenile and adult thirteen-lined ground squirrels and the role of nutritional need. Anim Behav 59: 535–541. 10715175 10.1006/anbe.1999.1345

[29] Magnhagen C (1991) Predation risk as a cost of reproduction. Trends Ecol Evol 6: 183–186. 21232452 10.1016/0169-5347(91)90210-O

[30] De Roos AM, Persson L, McCauley E (2003) The influence of size-dependent life history traits on the structure and the dynamics of populations and communities. Ecol Lett 6: 473–487.

[31] Boukal DS, Berec L, Křivan V (2008) Does Sex-Selective Predation Stabilize or destabilize predator-prey dynamics? PLoS ONE 3, 7,e2687.doi:10.1371/journal.pone.0002687. 10.1371/journal.pone.0002687 PMC2444021 18628951

[32] Steenbeek R, Piek RC, van Buul B, van Hooff ARAM (1999) Vigilance in wild Thomas's langurs (Presbytis thomasi): the importance of infanticide risk. Behav Ecol Sociobiol 45: 137–150.

[33] Burger J, Safina C, Gochfeld M (2000) Factors affecting vigilance in springbok: importance of vegetative cover, location in herd, and herd size. Acta Ethol 2: 97–104.

[34] Wilson JD, Evans J, Browne SJ, King JR (1997) Territory distribution and breeding success of skylarks Alauda arvensis on organic and intensive farmland in southern England. J App Ecol 34: 1462–1478.

[35] Chamberlain DE, Wilson AM, Browne SJ, Vickery JA (1999) Effects of habitat type and management on the abundance of skylarks in the breeding season. J App Ecol 36: 856–870.

[36] Donald PF, Green RE, Heath MF (2001) Agricultural intensification and the collapse of Europe's farmland bird populations. Proc R Soc Lond B 268: 25–29. 10.1098/rspb.2000.1325 PMC1087596 12123294

[37] Whittingham MJ, Devereux CL, Evans AD, Bradbury RB (2006) Altering perceived predation risk and food availability: management prescriptions to benefit farmland birds on stubble fields. J App Ecol 43: 640–650.

[38] Nystrand M (2006) Influence if age, kinship, and large-scale habitat quality on local foraging choices of Siberian jays. Behav Ecol 17: 503–509.

[39] Roberts G (1996) Why individual vigilance declines as group size increases. Anim Behav 51: 1077–1086.

[40] Beauchamp G (2009) How does food density influence vigilance in birds and mammals? Anim Behav 78: 223–231.

[41] Siriwardena GM, Baillie SR, Wilson JR (1998) Variation in the survival rates of some British passerines with respect to their trends on farmland. Bird Study 45: 276–292.

[42] Breitwisch R, Hudak R (1988) Sex differences in risk-taking behaviour in foraging flocks of House sparrows. The Auk 106: 150–153.

[43] Cramp S (1988) The Birds of the Western Palearctic, Vol. 5. Oxford University Press, Oxford.

[44] Chamberlain DE, Crick HQP (1999) Population declines and reproductive performance of skylarks Alauda arvensis in different regions and habitats of the United Kingdom. Ibis 141: 38–51.

[45] Eraud C, Lallemand J, Lormee H (2006) Sex-ratio of skylark Alauda arvensis in relation to timing of breeding: capsule earlier broods tend to be more male biased than later broods. Bird Study 53: 319–322.

[46] Robinson RA, Sutherland WJ (1999) The winter distribution of seed-eating birds: habitat structure, seed density and seasonal depletion. Ecography 22: 447–454.

[47] Moorcroft D, Whittingham MJ, Bradbury RB, Wilson JD (2002) The selection of stubble fields by wintering granivorous birds reflects vegetation cover and food abundance. J App Ecol 39: 535–547.

[48] Powolny T, Eraud C, Bretagnolle V (2012) Group size modulates time budget and foraging efficiency in captive Skylarks, Alauda arvensis. JOrnithol 153: 485–490.

[49] Fernandez-Juricic E, Kacelnik A (2004) Information transfer and gain in flocks: the effects of quality and quantity of social information at different neighbour distances. Behav Ecol Sociobiol 55: 502–511.

[50] Ottoni EB (1996) Etholog 1.0: Ethological transcription tool for Windows. Behavior Research Methods, Instruments and Computers 28: 472–473. 10.3758/bf03200814 11029818

[51] Whittingham MJ, Markland HM (2002) The influence of substrate on the functional response of an avian granivore and its implications for farmland bird conservation. Oecologia 130: 637–644. 28547267 10.1007/s00442-001-0850-z

[52] Fernadez-Juricic E, Siller S, Kacelnik A (2004) Flock density, social foraging and scanning: an experiment with starlings. Behav Ecol 15: 371–379.

[53] Crawley MJ (1993) GLIM for ecologists. Blackwell Scientific Publications, Oxford.

[54] Verdolin JL (2006) Meta-analysis of foraging and predation risk trade-offs in terrestrial systems. Behav Ecol Sociobiol 60: 457–464.

[55] Foster WA, Treherne JE (1981) Evidence for the dilution effect in the selfish herd from fish predation on a marine insect. Nature 295: 466–467.

[56] Smart SM, Stillman RA, Norris KJ (2008) Measuring the functional responses of farmland birds: an example of a declining seed-feeding bunting. J Anim Ecol 77: 687–695. 18577020 10.1111/j.1365-2656.2008.01375.x

[57] Cameron E, Du Toit JT (2005) Social influences on vigilance behaviour in giraffes, Giraffa camelopardalis. Anim Behav 69: 1337–1344.

[58] Ginnett TF, Demment MW (1997) Sex differences in giraffe foraging behavior at two spatial scale. Oecologia 110: 291–300. 28307437 10.1007/s004420050162

[59] Fitzgibbon CD (1990) Why do hunting cheetahs prefer male gazelles? Anim Behav 40: 837–845.

[60] Reboreda JC, Fernandez GJ (1997) Sexual, seasonal and group size differences in the allocation of time between vigilance and feeding in the greater rhea Rhea americana. Ethology 103: 198–207.

[61] McNamara JM, Houston AI (1990) State-dependent ideal free distributions. Evol Ecol 4: 298–311.

[62] Mills GL, Gorman ML (1997) Factors affecting the density and distribution of wild dogs in the Kruger national park. Conservation Biol 11: 1397–1406.

[63] Heithaus MR, Dill LM (2002) Food availability and tiger shark predation risk influence bottlenose dolphin habitat use. Ecology 83: 480–491.

[64] Cresswell W (1994b) Age-dependent choice of redshank (*Tringa tetanus*) feeding location: profitability or risk? J Anim Ecol 63: 589–600.

[65] Fernandez G, Lank DB (2010) Do sex and habitat differences in antipredator behaviour of Western Sandpipers *Calidris mauri* reflect cumulative or compensatory processes? J. Ornithol 151: 665–672.

[66] Pravosudov VV, Grubb TC (1995) Vigilance in the tufted titmouse varies independently with air temperature and conspecific group size. Condor 97: 1064–1067.

[67] Meldrum GE, Ruckstuhl KE (2009) Mixed-sex group formation by bighorn sheep in winter: trading costs of synchrony for benefits of group living. Anim Behav 77: 919–929.

[68] Lima SL (1998) Non-lethal effects in the ecology of predator-prey interactions. Bioscience 48: 25–34.

[69] Brabrand A, Faafeng B (1993) Habitat shift in roach (*Rutilus rutilus*) induced by pikeperch (*Stozestion lucioperca*) introduction: predation risk versus pelagic behaviour. Oecologia 95: 38–46. 28313309 10.1007/BF00649504

